# N_2_ fixation in free‐floating filaments of *Trichodesmium* is higher than in transiently suboxic colony microenvironments

**DOI:** 10.1111/nph.15621

**Published:** 2018-12-29

**Authors:** Meri Eichner, Silke Thoms, Björn Rost, Wiebke Mohr, Soeren Ahmerkamp, Helle Ploug, Marcel M. M. Kuypers, Dirk de Beer

**Affiliations:** ^1^ Max Planck Institute for Marine Microbiology Celsiusstr. 1 Bremen 28359 Germany; ^2^ Alfred Wegener Institute, Helmholtz Centre for Polar and Marine Research Am Handelshafen 12 Bremerhaven 27570 Germany; ^3^ Department of Marine Sciences University of Gothenburg Carl Skottbergsgata 22 B Göteborg 41319 Sweden

**Keywords:** colony, microenvironment, N_2_ fixation, oxygen, *Trichodesmium*

## Abstract

- To understand the role of micrometer‐scale oxygen (O_2_) gradients in facilitating dinitrogen (N_2_) fixation, we characterized O_2_ dynamics in the microenvironment around free‐floating trichomes and colonies of *Trichodesmium erythraeum* IMS101.
- Diurnal and spatial variability in O_2_ concentrations in the bulk medium, within colonies, along trichomes and within single cells were determined using O_2_ optodes, microsensors and model calculations. Carbon (C) and N_2_ fixation as well as O_2_ evolution and uptake under different O_2_ concentrations were analyzed by stable isotope incubations and membrane inlet mass spectrometry.
- We observed a pronounced diel rhythm in O_2_ fluxes, with net O_2_ evolution restricted to short periods in the morning and evening, and net O_2_ uptake driven by dark respiration and light‐dependent O_2_ uptake during the major part of the light period. Remarkably, colonies showed lower N_2_ fixation and C fixation rates than free‐floating trichomes despite the long period of O_2_ undersaturation in the colony microenvironment.
- Model calculations demonstrate that low permeability of the cell wall in combination with metabolic heterogeneity between single cells allows for anoxic intracellular conditions in colonies but also free‐floating trichomes of *Trichodesmium*. Therefore, whereas colony formation must have benefits for *Trichodesmium*, it does not favor N_2_ fixation.

## Introduction

Fixation of dinitrogen (N_2_) by marine diazotrophic bacteria and cyanobacteria provides a significant source of nitrogen to phytoplankton in oligotrophic systems. The N_2_‐fixing enzyme nitrogenase is inhibited by oxygen (O_2_) via oxidative damage to the iron sulfur clusters (Burgess & Lowe, 1996), proteolysis (Durner *et al*., 1996) as well as suppression of nitrogenase synthesis and posttranslational modification (Gallon, 1992). To protect nitrogenase from O_2_, many single‐celled cyanobacteria separate photosynthesis from N_2_ fixation in time, conducting N_2_ fixation during the night, whereas many filamentous cyanobacteria restrict N_2_ fixation to specialized cells termed heterocysts. The genus *Trichodesmium* spp., a globally important, colony‐forming diazotroph, is an exception in that it fixes N_2_ during the day although it lacks heterocysts. A range of O_2_‐protective mechanisms has been suggested for *Trichodesmium*: the formation of anoxic microzones within colonies (Paerl & Bebout, 1988); a downregulation of photosynthesis during the peak of N_2_ fixation at midday (Berman‐Frank *et al*., 2001); a restriction of N_2_ fixation to specialized cells termed diazocytes (Bergman & Carpenter, 1991; Berman‐Frank *et al*., 2001); and dynamic switches between different photosynthetic activity states by reversible uncoupling of phycobilisomes from the photosystems on the time scale of minutes (Küpper *et al*., 2004, 2009). The prevalence and coordination of these different mechanisms is still debated, partly due to conflicting results in previous studies.

Regarding cell specialization, several studies have reported nitrogenase to be present only in 10–15% of cells within a trichome (diazocytes), which are not terminally differentiated cells, do not have thicker cell walls than vegetative cells, and contain photosystem II, by contrast to heterocysts (Carpenter *et al*., 1990; Siddiqui *et al*., 1992a; Fredriksson & Bergman, 1995; Berman‐Frank *et al*., 2001). Other studies observed no differences in nitrogenase expression and/or N_2_ fixation between single cells and therefore questioned the prevalence of diazocytes (Paerl & Bebout, 1988; Finzi‐Hart *et al*., 2009; Ohki & Taniuchi, 2009). Also the formation of anoxic microzones in colonies has been challenged by field measurements showing strong O_2_ supersaturation in *Trichodesmium* colonies (Eichner *et al*., 2017), questioning the assumed benefit of colony formation for N_2_ fixation. Direct comparisons of colonies and single trichomes are scarce. Field studies have traditionally shown a bias towards studying colonies rather than single trichomes (Letelier & Karl, 1998), whereas most laboratory studies on *Trichodesmium* physiology, including those demonstrating the inhibitory effects of O_2_ on N_2_ fixation *in vivo* (Küpper *et al*., 2004; Berman‐Frank *et al*., 2005, 2008; Staal *et al*., 2007), have been conducted on cultures of *Trichodesmium erythraeum* IMS101 grown as single trichomes.

To re‐evaluate the hypothesis that anoxic microenvironments allow for higher N_2_ fixation in colonies compared with single trichomes, we conducted an in‐depth analysis of O_2_ dynamics and feedbacks on N_2_ fixation in colonies and free‐floating trichomes of *Trichodesmium erythraeum* IMS101. We directly compared carbon (C) and N_2_ fixation rates in colonies vs single trichomes and characterized cellular gross and net O_2_ fluxes, diurnal variations in O_2_ concentrations in the bulk medium, and O_2_ concentrations within colonies, on the surface of single trichomes and within single cells.

## Materials and Methods

### Culture conditions

Cultures of *Trichodesmium erythraeum* IMS101 were grown on YBCII culture medium (Chen *et al*., 1996) with decreased phosphate concentrations (4.61 ± 0.79 μmol l^−1^, determined with QuAAtro39, Seal Analytics), at 27–29°C and 240–280 μmol photons m^−2^ s^−1^ under a 12 h: 12 h, light : dark cycle. Stock cultures were grown on a shaking table (80 rounds  min^−1^, IKA KS 130 basic), and transferred to roller tanks once colonies had started to form in order to maintain colonies physically intact for longer. Additional colonies formed in roller tanks, including mostly puffs, but also tuft‐ and needle‐shaped colonies. Depending on the culture volume required for the specific measurements, different roller tank set‐ups were used, including bottles with a volume between 60 ml and 2.5 l. pH levels (National Bureau of Standards (NBS) scale) were determined with a two‐point calibrated glass electrode (Aquatrode plus Pt1000; Metrohm, Herisau, Switzerland).

### Elemental composition

For determination of elemental ratios, samples for particulate organic carbon, nitrogen and phosphorus (POC, PON and POP) and chlorophyll *a* (Chl*a*) were taken *c*. 7 h after beginning of the light phase. Technical duplicate samples of culture including both free trichomes and colonies were filtered onto precombusted GF/F filters and stored at −20°C. Samples for analysis of POC and PON were acidified with 200 μl HCl (0.2 mol l^−1^) and subsequently measured on an elemental analyzer (EuroEA, Eurovector). POP was determined spectrophotometrically (UV‐1202; Schimadzu, Kyoto, Japan) according to Hansen & Koroleff (1999). Chl*a* was extracted in 90% acetone at 4°C for > 12 h with ultrasonication (10 s) and measured fluorometrically (10‐AU, Turner Designs; Holm‐Hansen & Riemann, 1978).

### Optode measurements

The diurnal cycle of O_2_ concentrations in cultures was monitored with contactless optical O_2_ sensors glued into culture vessels (silicon glue, sensor spots of 5 mm diameter and a FireStingO2/FireStingGO2 oxygen meter, Pyroscience). Measurements were performed in 120 ml serum bottles closed without headspace and incubated in roller tanks. Additionally, O_2_ concentrations were monitored in the set‐up used for stock cultures, that is, culture vials closed with a gas permeable frit (VWR) that were incubated on a shaking table. O_2_ concentrations were recorded at 2 Hz for up to 7 d. Optodes were two‐point calibrated using medium that was bubbled with either N_2_ gas or air to achieve 0% and 100% air saturation, respectively.

### Microsensor measurements

Microsensor measurements on colonies were performed in a custom‐made flow system (Ploug & Jørgensen, 1999) in YBCII medium at 26°C. Single colonies were suspended in the flow chamber (flow < 0.1 mm s^−1^), fixed with a thin glass needle and observed through a stereomicroscope (Stemi SV6; Zeiss). For recording depth profiles through the colony center, microsensors were moved towards and into the colony with a motor‐driven micromanipulator (VT‐80, Micos/Faulhaber Minimotor SA).

In a total of 13 colonies, O_2_ concentrations in and around colonies were measured with Clark‐type microelectrodes (tip diameter *c*. 10–15 μm, response time *c*. 1 s). Rates of respiration and net photosynthesis were calculated from the steady‐state O_2_ gradient at the colony surface according to Fick's first law of diffusion:

J=−D(ΔC/Δr),

where J represents the interfacial O_2_ flux, D the diffusion coefficient for O_2_ (2.26 × 10^−9^ m^2^ s^−1^ at 25°C and salinity 34; Broecker & Peng, 1974), and ΔC the concentration difference measured over the respective distance, Δr, at the colony surface. Surface area and volume were determined for each colony assuming ellipsoid geometry and used to convert interfacial flux to volume‐normalized rates. The theoretical maximum O_2_ uptake supported by diffusive O_2_ supply (that is, the O_2_ uptake rate yielding anoxia at the center of the colony) was calculated as a function of colony volume as described by Ploug *et al*. (1997). An apparent diffusivity of O_2_ within the colony of 0.95× that of seawater, a Sherwood number of 1 (that is, no difference between the motion of the colony and that of the surrounding water, resulting in mass transfer merely by molecular diffusion but not advection), a bulk O_2_ concentration of 212 μmol l^−1^ (that is, air saturation), and a uniform respiration rate throughout the colony were assumed.

Additionally, measurements of chlorophyll fluorescence were performed on nine of the colonies using a MicrofiberPAM (Walz) with a microfiber tapered to 10–20 μm width at the tip. A red LED (650 nm) was used for excitation. Measurements were performed in the dark, between 1 and 9 h after beginning of the light phase. Photochemical quantum yield (*F*_v_/*F*_m_) is reported only for those positions in and around colonies for which signal strength was high enough for a clear fluorescence induction curve to be observed.

For microsensor measurements on single trichomes with higher spatial resolution, the trichomes were embedded in 0.5% agar, in which the effective diffusion coefficient of O_2_ is similar as in seawater (Ploug & Passow, 2007). Ultra‐pure low melting point agar (Invitrogen) was dissolved, allowed to cool down to < 30°C, and then mixed in a ratio of *c*. 1 : 1 with culture in a Petri dish. Measurements were performed on a total of 29 trichomes with Clark‐type microelectrodes (tip diameter 5–10 μm, response time *c*. 1 s) under an inverted microscope (Axiovert 25; Zeiss) at ×20 magnification, room temperature and *c*. 150 μmol photons m^−2^ s^−1^ (unless specified otherwise), at various time points between the start and 1 h after the end of the light period. Differences in O_2_ concentrations along trichomes were probed by moving the sensor along the trichome within *c*. 3 μm distance from the cell surface as observed in the microscope, and recording O_2_ concentrations in consecutive steps of three to four cells. Additionally, O_2_ concentrations were recorded continuously at the surface of individual cells while switching the light on and off.

### Membrane inlet mass spectrometry

Gross and net O_2_ fluxes were measured with a custom‐built membrane inlet mass spectrometer (MIMS; mass spectrometer Isoprime, custom‐built 8 ml cuvette) using the ^18^O_2_‐based approach described by Fock & Sültemeyer (1989). Cultures containing colonies and single trichomes were grown in 2.5 l Schott bottles at 25°C and 300 μmol photons m^−2^ s^−1^ (Biolux; Osram, Garching, Germany). For measurements, ^18^O_2_ gas (Chemotrade, Düsseldorf, Germany) was dissolved in previously N_2_‐bubbled YBCII medium buffered with HEPES (50 mmol l^−1^, pH 8.29 ± 0.06), reaching a final concentration of *c*. 150% air saturation. Cultures concentrated by gentle filtration over a polycarbonate filter, as well as additional colonies picked with a Pasteur pipette, were suspended in the ^18^O_2_‐enriched medium, reaching a final Chl*a* concentration of 0.41 ± 0.26 μg ml^−1^. The production of ^16^O_2_ and the uptake of ^16^O_2_ and ^18^O_2_ were then monitored in light and dark under three different O_2_ concentrations (346 ± 39 μmol l^−1^, 191 ± 51 μmol l^−1^ and 101 ± 35 μmol l^−1^) obtained by bubbling with N_2_ gas. Samples were stirred during measurements, resulting in disassembly of the colonies. Two replicate light and dark phases lasting *c*. 4 min each were conducted at each O_2_ level. O_2_ signals were corrected for abiotic consumption and influx of O_2_ into the cuvette by subtracting O_2_ slopes recorded in abiotic controls at the respective O_2_ concentrations. In total, 11 samples containing free‐floating trichomes and colonies in various proportions were analyzed. Measurements were performed between 1.5 and 4 h after the beginning of the light phase (except one measurement at 8–9 h after the beginning of the light phase that yielded results that were not different from the others).

### Stable isotope incubations

C and N_2_ fixation rates of free‐floating trichomes and colonies were determined by stable isotope incubations with NaH^13^CO_3_ (Sigma Aldrich) and ^15^N_2_ gas (Cambridge Isotope Laboratories, Tewksbury, MA, USA). To ensure dissolution of ^15^N_2_ gas, ^15^N_2_ and NaH^13^CO_3_ were predissolved in YBCII medium for > 12 h before incubations. Final enrichment was 3.44 ± 0.24 atom percent excess (at% excess) for ^13^C and 2.2 ± 1.06 at% excess for ^15^N, as determined at the end of incubations for each incubation bottle by cavity ring‐down spectroscopy (G2201‐i; Picarro, Santa Clara, CA, USA) and MIMS (GAM2000; InProcess, Bremen, Germany). O_2_ concentrations in the incubation medium were adjusted to low (24 ± 11 μmol l^−1^), ambient (238 ± 13 μmol l^−1^) or high (475 ± 32 μmol l^−1^) levels by bubbling with a mixture of helium and N_2_ (20% : 80%, low O_2_ treatment), room air (ambient O_2_ treatment), or a mixture of O_2_ and N_2_ (40% : 60%, high O_2_ treatment). Subsequently, *Trichodesmium* biomass was transferred to the respective medium and amended with ^15^N‐ and ^13^C‐enriched stock solutions. For incubations of colonies, approx. 50 colonies per incubation vial were picked from culture bottles with a Pasteur pipette. For incubations of free‐floating trichomes, the remaining culture volume was concentrated by filtration over a polycarbonate filter before transfer to the incubation medium. Samples where free‐floating trichomes formed colonies during the incubation period, resulting in colony‐dominated biomass, were classified as colonies. Incubations were performed in triplicate, in 60 ml serum vials at 25°C and 150 μmol photons m^−2^ s^−1^ (100–210 μmol photons m^−2^ s^−1^ in roller tanks), for 11 h starting within 1 h after beginning of the light period. The O_2_ concentration in each incubation vial was measured with O_2_ microsensors before and after incubations. Vials with single trichomes were gently agitated by hand *c*. 4 times over the incubation period. Vials with colonies were incubated in roller tanks. At the end of the incubation time, cultures were filtered on precombusted GF/F filters and stored at −20°C. Before analysis, filters were acidified in an HCl fume (overnight) to remove all inorganic carbon. Amounts of POC and PON and their isotopic composition were measured by elemental analyser isotope ratio mass spectrometry (EA‐IRMS, Delta Plus XP and Flash EA 112; Thermo Fisher Scientific, Waltham, MA, USA). ^13^C and ^15^N enrichment of the POC and PON relative to the bulk solution was then converted to C and N_2_ fixation rates normalized to cellular C and N biomass, respectively, yielding C‐ and N‐specific fixation rates. The dissolved inorganic carbon concentration in incubation vials at the end of the incubation period was never below 1820 μmol l^−1^.

## Results

### Culture characteristics

**Figure 1:**
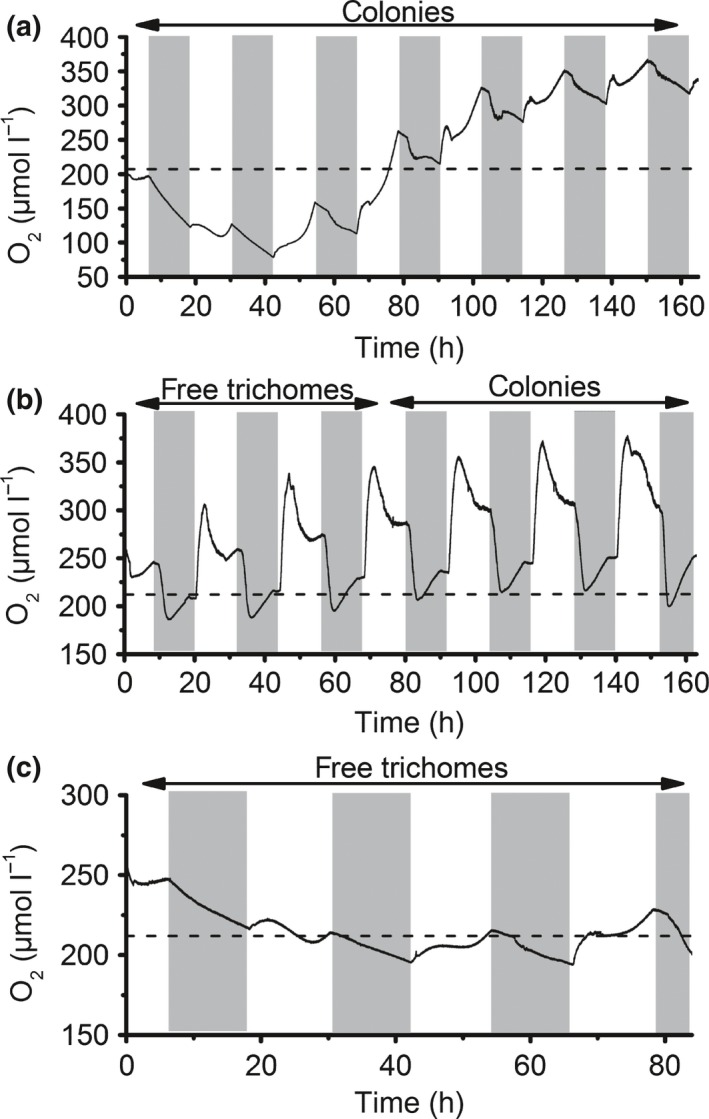
O_2_ concentrations in the bulk medium of *Trichodesmium* cultures incubated in roller tanks, monitored in three replicate vials (a–c). Dominant morphology (free‐floating trichomes or colonies) is indicated above each panel. Note that total biomass differed between vials and changed over time. Dashed lines indicate O_2_ concentration at air saturation, and grey shaded areas indicate dark phases (that is, night‐time).

Single trichomes and colonies co‐occurred in the cultures in varying proportions. Cultures generally started forming colonies 1 wk or longer after dilution with fresh medium and stayed in a colony‐dominated state for more than a week. Colonies were often formed in high numbers within a short time period, starting with needle‐ or string‐shaped colonies that formed around 200 mostly puff‐shaped colonies l^−1^ within a day, which were morphologically similar to field‐collected puffs. Continuous monitoring of bulk O_2_ concentrations within culture vials over several days showed that the cultures were net phototrophic most of the time (Fig. 1). This trend was independent of the relative abundance of colonies vs single trichomes, that is, no changes were observed once cultures went from a single‐trichome‐ to a colony‐dominated state (Fig. 1b). Key characteristics of the cultures are summarized in Table 1. pH levels were higher in cultures than in abiotic reference medium (*t*‐test, *P* < 0.005, Table 1), confirming net phototrophic growth. Monitoring of phosphate concentrations in two representative bottles over 8 d showed phosphate consumption. Elemental ratios in biomass were variable, with average values close to the Redfield ratio. POC : PON ratios in samples taken from stable isotope incubations at the end of the light period were higher than those taken *c*. 7 h after the beginning of the light period, and showed no significant difference between colonies and trichomes (*t*‐test, *P* > 0.05, Table 1).

**Table 1:** Key characteristics of *Trichodesmium* cultures used in the experiment (mean ± SD)

pH in culture medium		8.52 ± 0.06	*n* = 7
pH in abiotic reference medium		8.38 ± 0.06	*n* = 9
Phosphate uptake (replicate 1)	μmol l^−1^ d^−1^	0.08	
Phosphate uptake (replicate 2)	μmol l^−1^ d^−1^	0.12	
F_v_/F_m_ a		0.46 ± 0.07	*n* = 9
POC : PON (7 h)^b^	mol mol^−1^	5.5 ± 1.2	*n* = 4
POC : PON (12 h)^c^	mol mol^−1^	8.3 ± 2.6	*n* = 28
POC : POP^b^	mol mol^−1^	100 ± 75	*n* = 4
PON : POP^b^	mol mol^−1^	20 ± 16	*n* = 4
POC : chlorophyll *a* b	μg μg^−1^	256 ± 116	*n* = 3

F_v_/F_m_, photochemical quantum yield; POC, particulate organic carbon; PON, particulate organic nitrogen; POP, particulate organic phosphorus.

a Measured along transects across individual colonies.

b Bulk culture samples taken 7 h after beginning of the photoperiod.

c Samples taken at the end of the photoperiod, average value including colonies and single trichomes incubated under different O_2_ concentrations.

### Diel variation in bulk O_2_ concentrations

**Figure 2:**
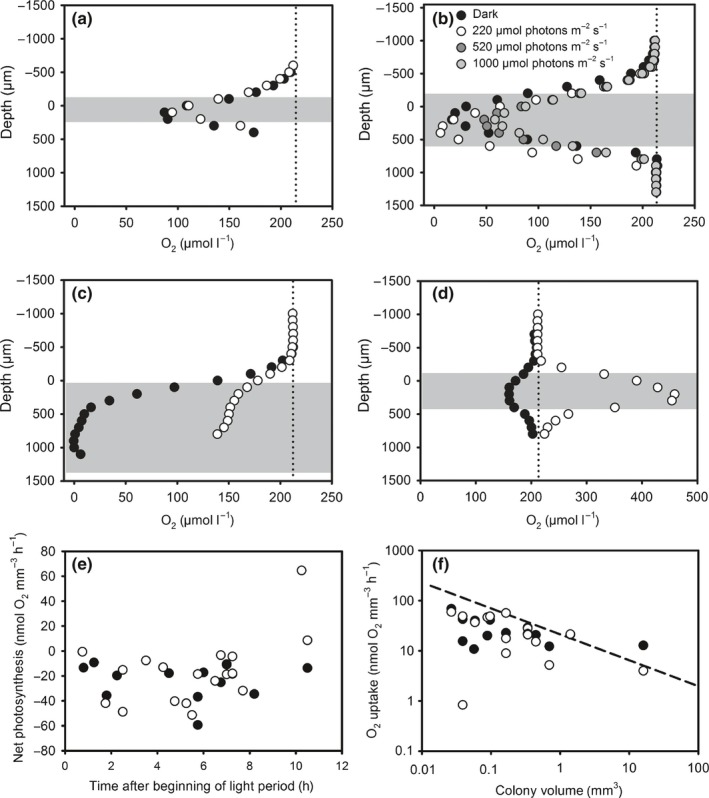
(a–d) Examples of O_2_ profiles measured in *Trichodesmium* colonies of different sizes (a, 0.02 mm^3^; b, 0.38 mm^3^; c, 2.4 mm^3^; d, 0.16 mm^3^) at different light intensities and times of the day (a, 6 h; b, 6 h; c, 7 h; d, 10 h, after the beginning of the light phase). Note the different scale of *x*‐axis. Grey shading indicates approximate position of the colony, dotted lines indicate O_2_ concentration at air saturation. (e) Net photosynthesis rates calculated from depth profiles through colonies measured at different times of the day. (f) Net O_2_ uptake by colonies in dependence of colony volume. Dashed line indicates diffusion limitation (calculated according to Ploug *et al*., 1997).

Continuous measurements of O_2_ concentrations in the culture medium revealed strong variations over the diel cycle, both in closed vials (Fig. 1), and in culture flasks with a gas permeable lid (up to 300 μmol l^−1^; data not shown). Often, a fast rise in O_2_ concentrations was observed within a short period of 1–3 h in the morning, followed by a slow decrease in O_2_ concentrations over several hours (Fig. 1). A second phase of net O_2_ evolution followed in most cases 2–4 h before the beginning of the dark phase. Net O_2_ evolution over the 12 h light period ranged between −3 μmol l^−1^ (12 h)^−1^ (that is, net O_2_ consumption) and 146 μmol l^−1^ (12 h)^−1^. During the night, a net decrease of O_2_ concentrations was observed, sometimes including a short and steep decrease during the first hours of the night. Remarkably, this initial decrease was followed by an increase to air saturation level or above, while the incubation was still in the dark (Fig. 1b). This pattern was confirmed by tracking O_2_ concentrations close to the center of single colonies with microsensors over several hours (Supporting Information Fig. S1b,d). In the morning, maximum O_2_ concentrations of 570 and 326 μmol l^−1^ were observed within colonies (0.5 and 1.3 h after beginning of the light period, respectively; Fig. S1a,c). In the evening, three out of six colonies examined showed elevated O_2_ concentrations (e.g. 450 μmol l^−1^ measured 2 h before the beginning of the dark period, Figs 2d, S1). During most of the day, however, colonies were undersaturated with O_2_ (Fig. 2a–c) and net photosynthesis calculated from O_2_ profiles was negative (Fig. 2e). To shed light on the regulation of O_2_ concentrations specifically during this phase of O_2_ undersaturation, O_2_ microenvironments and cellular gross and net O_2_ fluxes were analyzed in more detail during the middle of the day.

### O_2_ microenvironments in colonies during the middle of the day

O_2_ profiles were measured on a total of 13 colonies of different size (250–1500 μm diameter), shape (tufts and puffs), buoyancy and trichome density between 0.45 and 8 h after beginning of the light period, that is, covering the time period of decreasing O_2_ evolution rates in the bulk medium (Fig. 1). O_2_ concentrations in the center of the colony ranged from anoxic conditions to close to air saturation (Fig. 2a–c). Net O_2_ uptake rates ranged from 0.02 to 26.5 nmol colony^−1^ h^−1^. O_2_ uptake in light was correlated to dark respiration with an *R* ^2^ of 0.699 (linear regression). Volume‐normalized O_2_ uptake was not significantly affected by light intensity (*t*‐test, *P* > 0.05; Table 2) and colonies undersaturated with O_2_ were observed under light intensities up to 1000 μmol photons m^−2^ s^−1^ (Fig. 2b). A comparison of the measured O_2_ uptake rates with calculated O_2_ diffusion rates into the colony revealed that O_2_ uptake in both light and dark ranged up to the predicted maximum rate allowed by diffusive O_2_ supply (Fig. 2f). Measurements of chlorophyll fluorescence did not show any spatial patterns of photosynthetic activity along colony transects (data not shown).

**Table 2:** Net O_2_ uptake rates (mean ± SD) by *Trichodesmium* colonies based on O_2_ profiles measured at different light intensities at 45 min to 8 h after beginning of the light period

Light intensity	Net O_2_ uptake		
μmol photons m^−2^ s^−1^	nmol O_2_ colony^−1^ h^−1^	nmol mm^−3^ h^−1^	
Dark	4.0 ± 6.8	23 ± 14	*n* = 14
200	2.4 ± 2.3	24 ± 17	*n* = 16
500	1.4 ± 1.8	13 ± 8	*n* = 8
1000	3.1 ± 3.4	15 ± 1	*n* = 2

### Cellular O_2_ fluxes during the middle of the day

**Figure 3:**
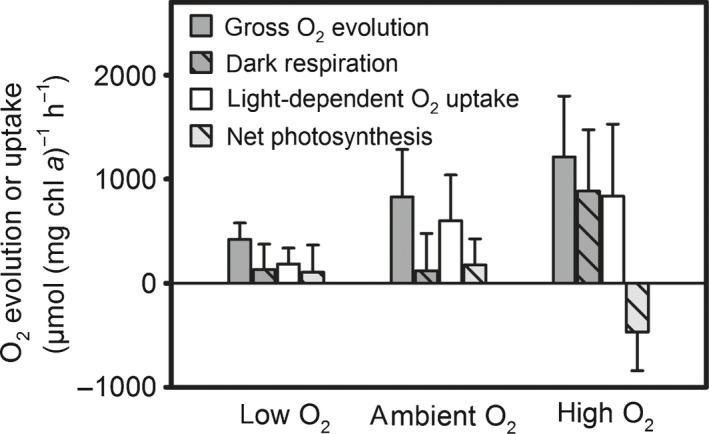
O_2_ production and uptake rates in *Trichodesmium* cultures measured during late morning/midday under different external O_2_ concentrations. Cultures contained both colonies and free trichomes. Note that positive values on the *y*‐axis indicate rates of both O_2_ evolving and O_2_ consuming processes. Light‐dependent O_2_ uptake is the difference between total O_2_ uptake measured in the light and total O_2_ uptake measured in the dark. Low O_2_, 101 ± 35 μmol l^−1^; ambient O_2_, 191 ± 51 μmol l^−1^; high O_2_, 346 ± 39 μmol l^−1^. *n *=* *11 for low and ambient O_2_, *n *=* *7 for high O_2_. Error bars ± SD.

Measurements of O_2_ fluxes in colonies and single trichomes during late morning/midday using an ^18^O_2_‐based MIMS approach yielded net O_2_ production rates that were often close to zero or negative (Fig. 3), in line with microsensor measurements performed during this part of the day (Fig. 2). The low net photosynthesis was a result of 4–5 times higher gross O_2_ uptake balancing gross O_2_ evolution. Dark respiration and light‐dependent O_2_ uptake (e.g. classical Mehler reaction and/or flavoprotein‐mediated O_2_ uptake) amounted to 56 ± 31% and 42 ± 63% of gross O_2_ evolution, respectively. Measurements under different external O_2_ concentrations mimicking the range observed in the center of colonies (e.g. Fig. 2) showed that gross O_2_ evolution, dark respiration, and light‐dependent O_2_ uptake increased with increasing external O_2_ concentration, whereas net photosynthesis decreased (analysis of variance (ANOVA), *P* < 0.05; Fig. 3) and was always negative when O_2_ concentrations above air saturation were applied. Test measurements revealed that these effects of elevated O_2_ levels were reversible; for instance, gross O_2_ evolution measured under ambient O_2_ was not significantly affected by O_2_ concentration in the preceding light phase (*t*‐test, *P* > 0.05, *n *≥* *4; data not shown). The relative amount of colonies and free‐floating trichomes did not have consistent effects on O_2_ fluxes, with higher dark respiration in colonies than free trichomes observed only under ambient O_2_ (*t*‐test, *P* < 0.05, *n *≥* *4) and higher total O_2_ uptake in colonies observed only under low O_2_ (*t*‐test, *P* < 0.05, *n *≥* *4).

### O_2_ on the surface of single trichomes

**Figure 4:**
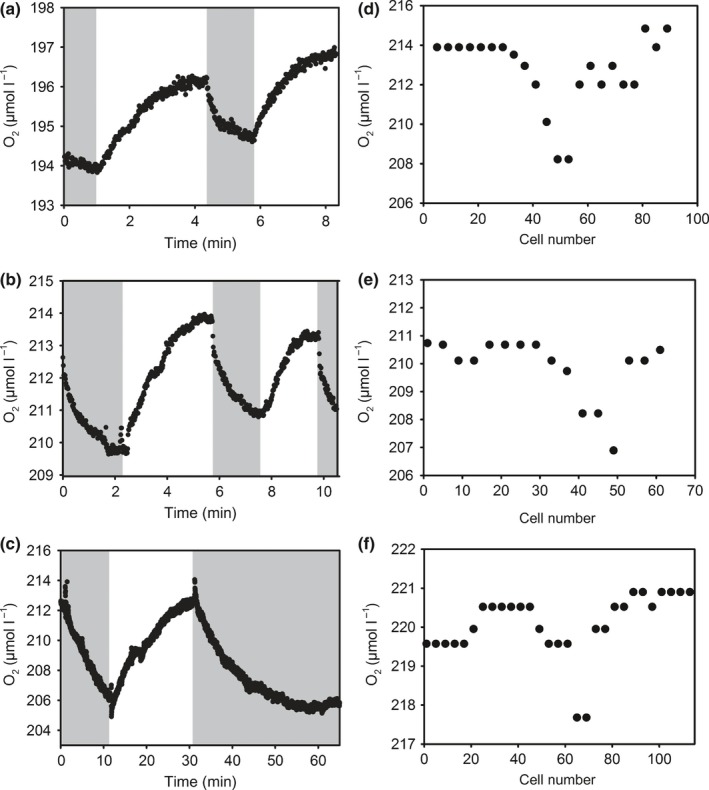
Examples of O_2_ concentrations measured within *c*. 3 μm of the surface of single *Trichodesmium* filaments during consecutive light and dark phases (a–c) and while sliding along the trichome with a microsensor (d–f; total duration of the measurement *c*. 20 min). Grey shaded areas indicate dark phases.

To analyze variability at the level of single cells, O_2_ concentrations on the surface of single trichomes were measured with microsensors at a spatial resolution equivalent to *c*. 2–4 cells (Fig. 4). O_2_ concentrations on the surface of single trichomes as well as in the surrounding agar never deviated more than *c*. 30 μmol l^−1^ from air saturation level. Changes in O_2_ concentrations on the cell surface when switching between light and dark ranged between 1 and 8 μmol l^−1^ (2.8 ± 1.8 μmol l^−1^, recorded in 27 measurements on a total of nine filaments; examples given in Fig. 4a–c). To test comparability of results from this set‐up with those obtained on colonies in the flow system, O_2_ concentrations were measured on the surface of a trichome located in the periphery of a tuft‐shaped colony, yielding a deviation in light vs dark of 70 μmol l^−1^ (data not shown). In the light, O_2_ concentrations on the trichome surface differed by up to 6 μmol l^−1^ between cells/regions within a single trichome (Fig. 4d–f). Distinct areas with lower than average O_2_ concentrations were observed in 11 out of the 26 trichomes analyzed. These areas were between 10 and 50 cells long and often located in the middle of trichomes.

### Effects of O_2_ concentrations on C and N_2_ fixation

**Figure 5:**
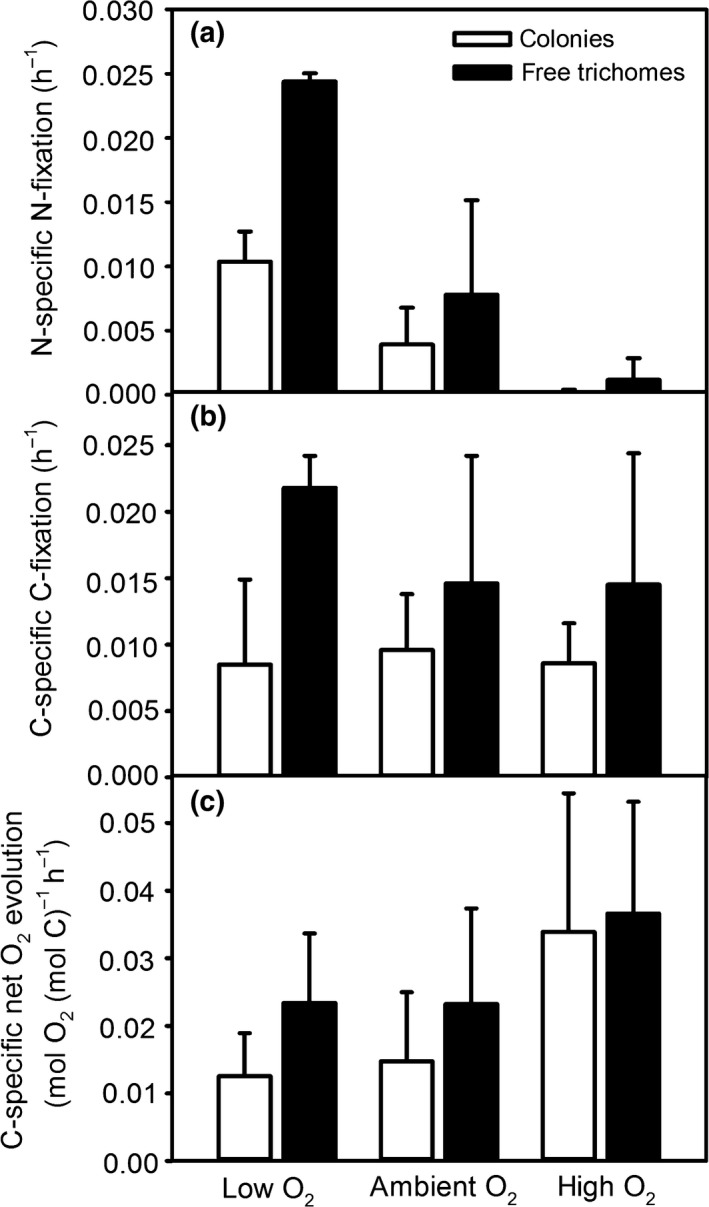
(a) N_2_ fixation, (b) C fixation and (c) O_2_ evolution in free‐floating trichomes and colonies of *Trichodesmium* measured under different O_2_ concentrations in the bulk medium over the duration of the light phase (12 h). Low O_2_, 24 ± 11 μmol l^−1^; ambient O_2_, 238 ± 13 μmol l^−1^; high O_2_, 475 ± 32 μmol l^−1^. *n *≥* *3, except for trichomes at low O_2_ with *n *=* *2 in (a, b), and colonies at high O_2_ with *n *=* *2 in (c). Error bars indicate SD.

N_2_ fixation was strongly O_2_‐dependent, with a clear inhibition in treatments with air‐saturated medium and medium containing elevated O_2_ concentrations compared to medium undersaturated in O_2_ (two‐way ANOVA and Holm‐Sidak test, *P* < 0.05; Fig. 5a). At low O_2_ concentrations, representative for minimum O_2_ levels observed in the colony center (e.g. Fig. 2), N_2_ fixation was increased by a factor of up to 4 compared to ambient O_2_ concentrations (Fig. 5a). At elevated O_2_ concentrations, representative for maximum O_2_ levels observed in the colony center (e.g. Fig. 2), N_2_ fixation rates were close to zero (Fig. 5a). Colonies showed lower N_2_ fixation rates than free‐floating trichomes (two‐way ANOVA, *P* < 0.05; Fig. 5a). Pair‐wise comparison under each O_2_ level showed that this effect was only significant under low O_2_ (Holm−Sidak test, *P* < 0.05). Similarly, colonies also showed lower C fixation rates than free‐floating trichomes (two‐way ANOVA, *P* < 0.05; Fig. 5b), with pair‐wise comparison showing a significant difference only under low O_2_ (Holm‐Sidak test, *P* < 0.05). C fixation was not significantly affected by the O_2_ treatment (two‐way ANOVA, *P* > 0.05). C‐specific O_2_ evolution was not significantly affected by the O_2_ treatment and was not different between colonies and free‐floating trichomes (two‐way ANOVA, *P* > 0.05; Fig. 5c).

## Discussion

### Physiological state of the cultures

Several observations indicate net biomass production in the colony‐forming cultures in this study. Firstly, the increases in pH and phosphate consumption in the medium demonstrate that the cultures were growing (Table 1). Although brief net heterotrophic phases were observed (e.g. first day in Fig. 1a,c), which could potentially be sustained by uptake of dissolved organic matter accumulated in the medium (Benavides *et al*., 2017), O_2_ concentrations generally increased over the duration of several days, confirming net autotrophy (Fig. 1). Both free‐floating trichomes and colonies were able to significantly increase N_2_ fixation rates within a day when exposed to reduced O_2_ concentrations (Fig. 5a). Colonies formed during the 12 h incubation period had similar N_2_ fixation rates as older colonies (data not shown), indicating that ‘old’ colonies were not in a senescent state. These findings are in contrast with a previous report attributing low N_2_ fixation rates in colonies of *Trichodesmium* NIBB 1067 to colony formation only during stationary growth phase (Ohki & Fujita, 1988).

C : N and C : Chl*a* ratios in particulate organic matter as well as C and N_2_ fixation rates were in a similar order of magnitude as previous data on field‐collected *Trichodesmium* colonies (Letelier & Karl, 1998; Eichner *et al*., 2017). POC : POP ratios were lower than those previously measured in the field (Letelier & Karl, 1998; Orcutt *et al*., 2013), and similar to P‐replete cultures of *Trichodesmium* IMS101 (Spungin *et al*., 2014), indicating that our cultures were not P‐limited. *F*_v_/*F*_m_, a general indicator for the health status of photosynthetic organisms that is often used to detect iron (Fe) limitation, was higher in our study than previous measurements on free‐floating, exponentially growing trichomes of the same strain (0.44 ± 0.11 (our study) vs 0.18–0.35 (Eichner *et al*., 2014; measured with a blue LED)), suggesting that colonies were not stressed or severely Fe‐depleted. By contrast with a recent laboratory study by Tzubari *et al*. (2018), we therefore assumed that, in our experiment, colony formation was not induced by P‐ or Fe‐depletion. As we did not observe any differences in abiotic conditions or cellular composition between free‐floating trichomes and colonies, the triggers for colony formation could not be identified. As cultures were grown under the same (*macro*scale) conditions, we relate the observed physiological differences to microenvironments within colonies.

### Variation over the diel cycle

The downregulation of net photosynthesis during the middle of the day (Figs 1, 2e) is in line with previous observations on *Trichodesmium* in the field and laboratory (Berman‐Frank *et al*., 2001; Eichner *et al*., 2014) and coincides with the peak in N_2_ fixation observed 4–5 h after the beginning of the light period in *Trichodesmium* IMS101 (e.g. Kranz *et al*., 2010; Eichner *et al*., 2014). The switch from N_2_ fixation back to photosynthesis in the afternoon was also reflected in higher POC : PON ratios measured at the end of the light period compared with the afternoon (Table 1), in line with previous observations (Kranz *et al*., 2009). The magnitude of diel variations in O_2_ was exceptionally large, reaching net O_2_ uptake during the middle of the day (Figs 1, 2e), whereas previous studies merely showed a reduction of net O_2_ evolution by 40–70% (Berman‐Frank *et al*., 2001; Eichner *et al*., 2014). To understand the physiological mechanisms leading to this net O_2_ uptake during the N_2_ fixation phase, we investigated gross O_2_ fluxes in this period by MIMS.

These measurements revealed a high ratio of gross to net O_2_ evolution comparable with previous field observations on *Trichodesmium* (e.g. Kana, 1993), and exceeding previous estimates for free‐floating trichomes of *Trichodesmium* IMS101 under comparable conditions (Kranz *et al*., 2010; Eichner *et al*., 2014). Such high O_2_ uptake suggests that *Trichodesmium* decreases O_2_ concentrations during the day by an upregulation of O_2_‐consuming processes such as dark respiration, classical Mehler reaction and/or flavoprotein‐mediated O_2_ uptake, rather than a downregulation of O_2_ evolution. This way, electron transport through the photosynthetic and respiratory transport chain, and therefore proton translocation, is maintained even through the phase of low net O_2_ production. This may serve as a means to maintain the production of ATP to support N_2_ fixation, and is also in line with the relatively high F_v_/F_m_ observed at the same time (Table 1). While O_2_ uptake by Mehler reaction does not involve carbon fluxes, the high dark respiration rates observed here require the breakdown of a significant amount of carbohydrates produced in the preceding morning hours. For instance, maintaining the dark respiration rates observed at high O_2_ concentrations (Fig. 3) for 7 h would consume *c*. 30% of the POC at a typical cell density observed in our cultures (200 μmol C l^−1^). In *Crocosphaera*, a similar diel cycle including the build‐up of carbohydrates during the day and their breakdown at night has been proposed to support the high energy demand related to protecting nitrogenase under ambient O_2_ levels (Großkopf & LaRoche, 2012). In *Trichodesmium*, energy and carbon budgets may differ substantially on a single‐cell level due to cell specialization, depending on how diazocytes acquire carbohydrates and/or energy equivalents to support respiration and N_2_ fixation. As the downregulation of net photosynthesis under elevated O_2_ observed during MIMS measurements in the late morning (Fig. 3) was not observed in 12 h incubations (Fig. 5c), it seems to be a transient effect that is outbalanced on a diurnal time scale. It might represent a regulatory response that induces a decrease in O_2_ concentrations once a certain threshold is reached during the accumulation of O_2_ in the phase of high O_2_ evolution in the morning. Such a switch in metabolism towards higher O_2_ uptake would ultimately lead to transient O_2_ undersaturation (Fig. 2a–c) within colonies, allowing for N_2_ fixation to start during midday.

### Impacts of colony formation

Colony formation resulted in pronounced microenvironments with O_2_ undersaturation during a large part of the day, reaching nearly anoxic conditions in the center of some of the colonies (Fig. 2a–c). However, as colonies in this study were mostly net phototrophic over a period of several days (Fig. 1), the long phase of O_2_ undersaturation must have been outbalanced by a *c*. 10 times shorter phase of strong O_2_ supersaturation. Indeed, microsensor measurements revealed O_2_ concentrations within colonies of up to 570 μmol l^−1^ (Figs S1, 2d). Even under air‐equilibrated bulk conditions, O_2_ concentrations within colonies therefore temporarily exceeded those supplied in the high O_2_ treatment, where N_2_ fixation was completely inhibited (Fig. 5a). However, this inhibitory effect of O_2_ on N_2_ fixation was reversible, as N_2_ fixation rates could be increased by exposure to low O_2_ concentrations (Fig. 5a), in line with previous studies showing recovery of nitrogenase after exposure to high O_2_ (Zehr *et al*., 1993). Hence, the lowered N_2_ fixation in colonies compared with single trichomes is most likely not caused by damage to nitrogenase during the phase of high O_2_ accumulation in the morning.

However, elevated O_2_ concentrations may lead not only to inactivation or degradation of O_2_‐sensitive proteins such as nitrogenase, but also induce damage to other cellular components relevant for both N_2_ fixation and C fixation, including lipids, proteins and DNA, through a concurrent increase in reactive oxygen species such as superoxide (e.g. Lesser, 2006). In line with this, high superoxide production by field‐collected *Trichodesmium* colonies has been observed (Hansel *et al*., 2016). Accumulation of O_2_ and reactive oxygen species in the colony microenvironment may therefore increase the risk of oxidative stress in colonies compared with single trichomes and might be part of the reason why colonies showed lower N_2_ and C fixation rates. The high rates of both gross O_2_ evolution and O_2_ uptake observed under elevated O_2_ concentrations (Fig. 3) might place additional strain on the turnover of the photosynthetic machinery during the periods of elevated O_2_ concentrations in colonies.

Moreover, the high rates of photosynthesis during the morning and evening (Fig. 1) may lead to a temporary depletion of carbon dioxide (CO_2_) within the colony microenvironment. In line with this, a correlation of ^13^C composition with colony size has been observed in field‐collected *Trichodesmium* colonies, which may reflect cellular responses to CO_2_ limitation in larger colonies (Tchernov & Lipschultz, 2008). In combination with the concurrently elevated O_2_ concentrations, CO_2_ depletion is likely to induce photorespiration and therefore lower C fixation rates. While CO_2_ depletion can be partly compensated by carbon concentrating mechanisms (CCMs) such as the active uptake of bicarbonate (HCO_3_^−^), the elevated energy expenditure for CCMs poses an additional disadvantage for colonies compared with free‐floating trichomes. Diffusion limitation in the colony microenvironment therefore involves several negative effects that may counteract the benefits of temporary O_2_ depletion for N_2_ fixation.

Our combined measurements of N_2_ fixation rates and small‐scale O_2_ gradients contradict the hypothesis of colony formation as a mechanism to foster N_2_ fixation by protecting nitrogenase from O_2_ (Paerl & Bebout, 1988). Instead, we were able to demonstrate that O_2_ undersaturation in the microenvironment (Fig. 2a–c) during the phase of typically highest nitrogenase activity (e.g. Berman‐Frank *et al*., 2001; Eichner *et al*., 2014) did not result in elevated N_2_ fixation rates in colonies (Fig. 5a). Consequently, we suggest that previous observations of higher or similar N_2_ fixation rates in colonies compared with free‐floating trichomes in the field (Saino & Hattori, 1982; Letelier & Karl, 1998) were not due to O_2_ microenvironments, but due to other factors not included in our laboratory setting. These might include positive effects of colony formation on the nutritional status of *Trichodesmium* that are manifested only under nutrient‐limited conditions in the field, such as enhanced dust dissolution (Rubin *et al*., 2011) and more efficient interaction with bacterial associates producing siderophores and/or alkaline phosphatase (Chappell & Webb, 2010; Orcutt *et al*., 2013; Lee *et al*., 2017). By excluding these additional factors in our laboratory study under nutrient‐replete conditions, we revealed that O_2_ microenvironments *per se* did not lead to higher N_2_ fixation rates in colonies compared with free‐floating trichomes. In addition to microbial interactions and higher nutrient (P and Fe) availability, reduced grazing and efficient vertical movement along light and nutrient gradients (e.g. reviewed by Beardall *et al*., 2009; Stal, 2017) may contribute to making colony formation a selective advantage in natural ecosystems.

### Intracellular O_2_ concentrations

Ultimately, nitrogenase activity is controlled by intracellular O_2_ concentrations in the vicinity of the protein rather than extracellular O_2_ conditions. On the cell surface of single trichomes, O_2_ concentrations were never below 180 μmol l^−1^. Hence, assuming that anoxic conditions are required in the vicinity of nitrogenase for it to function, cells must be able to establish a large gradient in O_2_ concentrations across the cell wall. If cells are relatively impermeable, O_2_ concentrations may differ strongly between neighboring cells in a trichome, depending on their specific metabolic activity. Our analysis of cell surface O_2_ concentrations on a single‐cell level revealed regions with reduced O_2_ concentrations (Fig. 4b). While we cannot exclude that these are senescing or dividing cells, it is interesting to note that their occurrence and preferential localization in the center of single trichomes is also in line with the proposed concept of transiently or permanently nonphotosynthetic diazocytes (e.g. Lin *et al*., 1998; Berman‐Frank *et al*., 2001).

To examine variation in intracellular O_2_ in more detail, we modeled the relation between membrane permeability, intracellular O_2_ concentrations and respiratory O_2_ fluxes based on our data, using a model described in Damm *et al*. (2015; Notes S1). Previous studies examining the interplay of O_2_ diffusion, consumption and N_2_ fixation in *Trichodesmium* assumed either no or very low (1–7 × 10^−1^ m s^−1^) diffusion resistance to O_2_ for *Trichodesmium* cells (Staal *et al*., 2003; Milligan *et al*., 2007). Here, we chose a substantially different approach in that permeability of the cell wall was the ultimate model output. Assuming that nitrogenase can only function under locally anoxic conditions, we calculated the permeability that would be necessary to yield an O_2_ concentration close to zero in the center of a diazocyte, based on cell dimensions and respiration rates measured by MIMS (Table 3). Since, strictly speaking, respiration cannot be sustained under anoxic conditions, the optimum intracellular O_2_ concentration is most likely to be slightly above zero, and our model output represents a conservative estimate of the required diffusion resistance. As histological studies on *Trichodesmium* indicated that thylakoids are distributed relatively homogenously in the cytosol (Siddiqui *et al*., 1992a) we assumed that there was uniform O_2_ consumption throughout the cell.

**Table 3:** Calculated permeability of the cell wall necessary to support anoxia in the interior of a *Trichodesmium* cell, based on measured rates of O_2_ uptake (that is, dark respiration), extracellular O_2_ concentrations in colonies and cell dimensions (average 5 μm length, 7 μm width)

Respiration distribution	External O_2_ (μmol l^−1^)	Average O_2_ uptake (fmol cell^−1^ h^−1^)	Intensity of O_2_ consumption (mol s^−1^ m^−3^)	Permeability for O_2_ (m s^−1^)	Permeability for CO_2_ (m s^−1^)
Homogeneous	329	608	1.49	4.6 × 10^−6^	3.7 × 10^−6^
Heterogeneous	329	3648	8.96	2.9 × 10^−5^	2.3 × 10^−5^
Homogeneous	187	194	0.48	2.6 × 10^−6^	2.1 × 10^−6^
Heterogeneous	187	1164	2.86	1.6 × 10^−5^	1.3 × 10^−5^
Homogeneous	101	198	0.49	4.9 × 10^−6^	3.9 × 10^−6^
Heterogeneous	101	1188	2.92	3.1 × 10^−5^	2.4 × 10^−5^

Scenarios with heterogeneous distribution of respiration assume a six‐fold higher respiration rate in diazocytes compared to vegetative cells. Permeability for CO_2_ was calculated from O_2_ permeability using a conversion factor of 0.7961 according to Ramsing & Gundersen (1994). For further details on the model, please refer to Supporting Information Notes S1.

Our model calculations revealed that single cells can achieve anoxic conditions in the center with a cell wall permeability for O_2_ between 2.6 × 10^−6^ and 4.9 × 10^−6^ m s^−1^ if they perform dark respiration at the average rate observed in this study (homogenous respiration, Table 3). Based on a previous study demonstrating single‐cell variation in cytochrome oxidase content that was correlated to nitrogenase content in *Trichodesmium thiebautii* (Bergman *et al*., 1993), we also considered the effects of a six‐fold higher respiration rate in diazocytes compared with vegetative cells, yielding somewhat higher permeability estimates between 1.6 × 10^−5^ m s^−1^ and 3.1 × 10^−5 ^m s^−1^ (heterogeneous respiration, Table 3). These estimates exceed a previous one for heterocysts (4 × 10^−7^ m s^−1^; Walsby, 1985), in line with the fact that *Trichodesmium* cell walls lack the thick glycolipid layer that acts as a diffusion barrier in heterocysts. In contrast with eukaryotic cells, plasma membranes of *Trichodesmium* are surrounded by a peptidoglycan layer, which was also reported to be more pronounced than in other Gram‐negative bacteria (Siddiqui *et al*., 1992b), and can explain why our estimates for CO_2_ permeability (calculated from O_2_ permeability according to Ramsing & Gundersen, 1994; Table 3) are lower than estimates for diatoms (10^−3^ m s^−1^; Hopkinson *et al*., 2011). Carboxysomes, in turn, with their protein shell acting as a diffusion barrier to CO_2_ (e.g. Rae *et al*., 2013), are expected to have a lower permeability to CO_2_ than the plasma membrane and cell wall. Accordingly, our estimates for CO_2_ permeability exceeded previous estimates for the cyanobacterium *Synechococcus* that were based on measurements of cellular CO_2_ efflux and therefore represent an integrated estimate for the cell wall and carboxysome (10^−6^–10^−7^ m s^−1^, Badger *et al*., 1985; 10^−8^ m s^−1^, Salon *et al*., 1996).

As there are no indications for differences in membrane permeability between individual *Trichodesmium* cells, anoxic conditions in diazocytes must be accompanied by O_2_ accumulation in photosynthesizing cells. Hence, while low permeability favors N_2_ fixation in diazotrophic (nonphotosynthetic) cells, it also increases the risk for photorespiration and oxidative stress in vegetative (photosynthetic) cells. Applying membrane permeabilities between 2.6 × 10^−6^ and 4.9 × 10^−6^ m s^−1^ (calculated for diazocytes as described above; Table 3) and a photosynthesis rate of 1013 fmol cell^−1^ h^−1^ (based on an O_2_ profile measured 10 h into the light period; Fig. 2d), we calculated intracellular O_2_ concentrations representative for the net O_2_ evolution phase in the morning/evening, yielding concentrations between 623 and 1166 μmol l^−1^ (Table 4). Intracellular O_2_ concentrations can therefore differ strongly between single cells, depending on their instantaneous rates of photosynthesis and respiration. These rates may change significantly not only over the diel cycle but potentially also as cells switch between N_2_‐fixing and non‐N_2_‐fixing states. The unexpected, transient nighttime increase in O_2_ concentrations observed with microsensors within colonies (Fig. S1) and with optodes in the bulk medium in closed vials (Fig. 1b) furthermore suggests that intracellular O_2_ dynamics may additionally be influenced by intracellular O_2_ reservoirs and/or chemical binding of O_2_, which should be targeted in future research.

**Table 4:** Calculated intracellular O_2_ concentrations in *Trichodesmium* under net O_2_ producing conditions (representative of early morning or evening), based on measured rates of O_2_ production, extracellular O_2_ concentrations in colonies, cell dimensions (5 μm length, 7 μm width), and membrane permeability calculated for diazocytes with an anoxic cell interior (Table 3)

Net O_2_ production (fmol cell^−1^ h^−1^)	External O_2_ concentration (μmol l^−1^)	Intensity of O_2_ production (mol s^−1^ m^−3^)	Permeability for O_2_ (m s^−1^)	Intracellular O_2_ concentration (μmol l^−1^)
1031	329	2.53	4.6 × 10^−6^	884
1031	187	2.53	2.6 × 10^−6^	1166
1031	101	2.53	4.9 × 10^−6^	623

Cell‐specific O_2_ production was calculated from a colony‐specific measurement assuming 10 000 cells per colony. For further details on the model, please refer to Supporting Information Notes S1.

Notably, our model calculations demonstrate that O_2_ undersaturation on the cell surface is not a prerequisite for anoxic conditions in diazocytes. In fact, anoxic conditions could be achieved within nonphotosynthetic cells even under elevated ambient O_2_ concentrations (Table 3). Colony formation as a means to form anoxic microenvironments is therefore not required, explaining why also single trichomes can fix N_2_ at high rates (Fig. 5a). More generally, our model results highlight that due to the low permeability of the cell wall, intracellular O_2_ concentrations in *Trichodesmium* are largely determined by cellular O_2_ fluxes rather than extracellular microenvironments. Importantly, extracellular O_2_ microenvironments can, however, indirectly affect intracellular conditions by inducing physiological responses of cellular O_2_ fluxes to external O_2_ concentrations (Fig. 3).

### Conclusions

**Figure 6:**
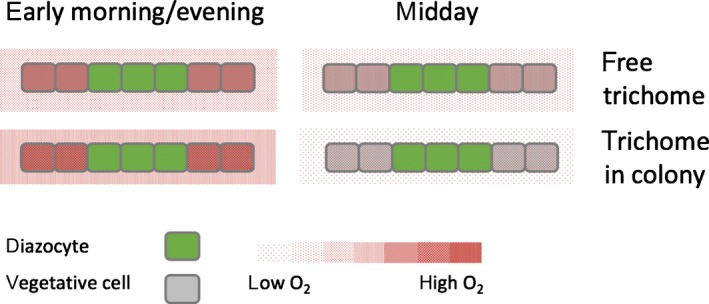
Conceptual model of intra‐ and extracellular O_2_ concentrations in *Trichodesmium* at different times of the day. Note that (nonphotosynthetic) diazocytes are always anoxic, while O_2_ concentrations in vegetative (photosynthetic) cells vary with time of the day and morphology (colony vs free trichomes). Estimates are based on O_2_ measurements (by microsensors and membrane inlet mass spectrometry (MIMS)) and model calculations, assuming the same photosynthesis rate and permeability for all cells. Red pattern in and around cells indicates O_2_ concentration, grey and green backgrounds indicate vegetative cells and diazocytes, respectively.

In summary, our measurements and model calculations demonstrate strong variations in O_2_ concentrations in colony microenvironments and also indicate large variations within single cells (Fig. 6). These were induced by changes in the metabolic activity of *Trichodesmium* over the diel cycle, including high O_2_ uptake during midday. Although colonies were undersaturated with O_2_ during most of the day, this did not result in higher N_2_ fixation rates by colonies compared with single trichomes, potentially as the O_2_ undersaturation over part of the day was accompanied by high extra‐ and intracellular O_2_ concentrations during a short time window in the morning and evening. Based on our model calculations and high resolution microsensor measurements, we hypothesize that the diffusion barrier provided by the cell wall in combination with physiological heterogeneity between single cells allows for anoxic conditions within diazocytes, that is, respiring, nonphotosynthetic cells, under air‐saturated bulk conditions (Fig. 6). Hence, O_2_ undersaturation in colony microenvironments is not required to support N_2_ fixation. Future research should therefore focus on the benefits of colony formation in *Trichodesmium* that outweigh the negative effects on C and N_2_ fixation.

## Author contributions

ME, BR, WM and DdB designed the study, ME and SA performed the experiments and analyzed the data, ST performed the model calculations, ME, ST, BR, WM, SA, HP, MMMK and DdB discussed data interpretation and wrote the manuscript.

## Supporting information

Please note: Wiley Blackwell are not responsible for the content or functionality of any Supporting Information supplied by the authors. Any queries (other than missing material) should be directed to the *New Phytologist* Central Office.

**Fig. S1** O_2_ concentrations recorded within *Trichodesmium* colonies over several hours.
**Notes S1** Calculation of the diffusion resistance to oxygen.
