# Supplementary material for: N2 fixation in free‐floating filaments of Trichodesmium is higher than in transiently suboxic colony microenvironments
Source: New Phytol. 2018 Dec 29;222(2):852–63. doi: 10.1111/nph.15621 (PMC6590460; doi:10.1111/nph.15621)
Supplement: Supplementary file 1 — Fig. S1 O2 concentrations recorded within Trichodesmium colonies over several hours. Notes S1 Calculation of the diffusion resistance to oxygen. [file NPH-222-852-s001.pdf]

***New Phytologist* Supporting Information**

Article title: N_2 fixation in free-floating filaments of *Trichodesmium* is higher than in transiently suboxic colony microenvironments

Authors: Meri Eichner, Silke Thoms, Björn Rost, Wiebke Mohr, Soeren Ahmerkamp, Helle Ploug, Marcel MM Kuypers and Dirk de Beer

The following Supporting Information is available for this article:

Fig. S1 O_2 concentrations recorded within *Trichodesmium* colonies over several hours.

Notes S1 Calculation of the diffusion resistance to oxygen.

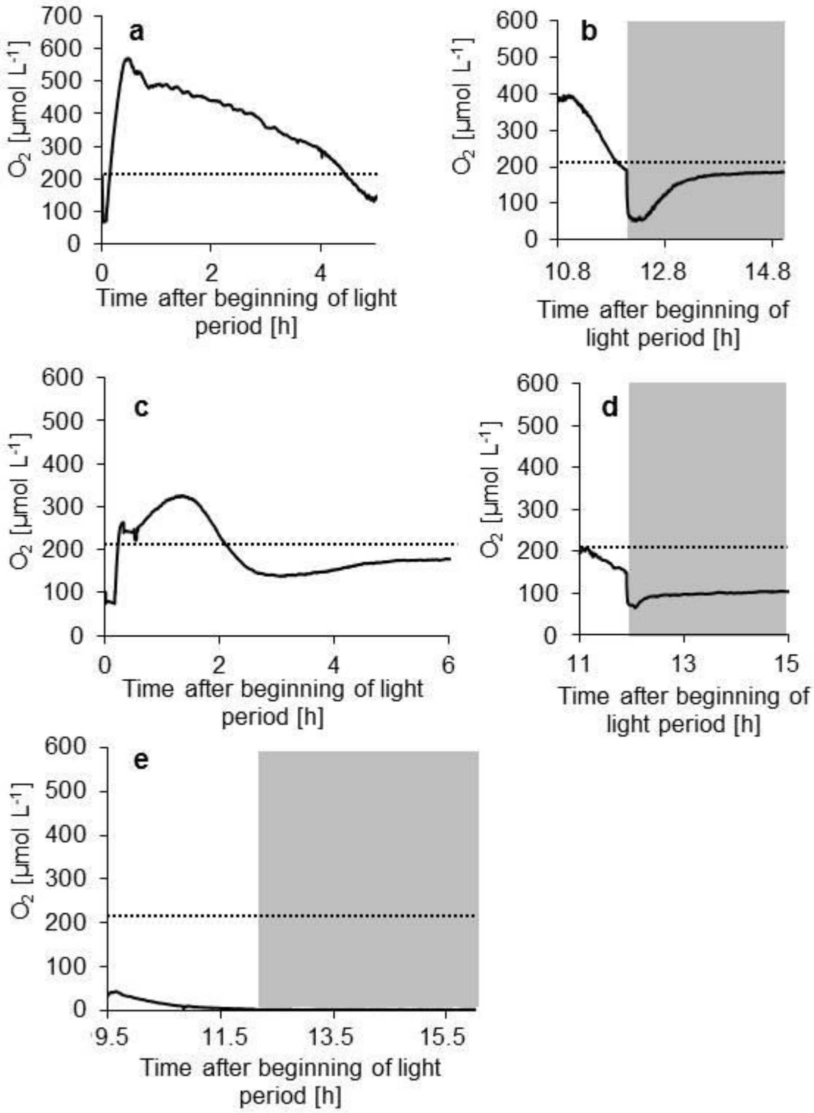

Fig. S1: O_2 concentrations recorded within *Trichodesmium* colonies over several hours. The sensor was kept at the same position, close to the center of the colony, over the duration of the measurement. Panels **a** to **e** show measurements on five different replicate colonies. Dotted lines indicate O_2 concentration at air saturation, grey shaded areas indicate dark phases (i.e., night time).

Notes S1: Calculation of the diffusion resistance to oxygen

As nitrogenase activity is not favoured in aerobic environment it is assumed that N_2 fixation by *Trichodesmium* occurs in microenvironments within the cells, which are sufficiently lacking in molecular oxygen. Here, we apply the model described in Damm et al. (2015) to calculate the oxygen concentration profile within single cells of *Trichodesmium* as a function of the cell properties (cell size, rates of respiratory metabolism, membrane permeability for O_2) and the external O_2 concentration. In order to keep the model simple, we describe the cell in terms of a sphere covered by a thin membrane. For this purpose, we use the 'surface area equivalent radius', *b*, which is defined as the radius of a hypothetical spherical cell with the same surface area as the more realistic cell shape (details of the method are described in Wolf-Gladrow & Riebesell, 1997). A *Trichodesmium* cell is treated as a circular cylinder with radius R = 3.5µm and length l = 5µm, which is in contact with the adjacent cells over the circular base surfaces and with the external medium over the lateral surface of the cylinder. Assuming that gas exchange between the cell and the external medium takes place via the lateral area, *b* is defined by

$$4\pi b^2 = \text{lateral area of the cell} = 2\pi R l. \qquad (1)$$

Using the values for R and l of *Trichodesmium*, it follows from equation (1): b = 2.96µm (for the calculations in Table 3 and Table 4 we used b = 3µm). Here, *b* is defined as the radius of the cell excluding the cell membrane. The membrane itself is described by a homogeneous spherical shell of outer and inner radii *a* and *b*. Within the interior of the sphere there is an O_2 consumption of constant intensity ρ. To determine the stationary concentration profile in the interior of the sphere we have to find the solution in the region $0 \leq r \leq b$ of

$$D\frac{1}{r^2}\frac{\mathrm{d}}{\mathrm{d}r}\left(r^2\frac{\mathrm{d}C(r)}{\mathrm{d}r}\right)-\rho(r)=0\,, \tag{2}$$

where D is the diffusion coefficient of O_2 and $C(r)$ is the concentration of O_2 as a function of the radial distance r from the centre of the cell. Assuming a free-floating cell, the diffusion coefficient in the surrounding water (D_w) is a constant and is given by the value in bulk seawater with a salinity of 34 at 25°C. Inside the cell, the salinity is probably slightly lower than in the surrounding seawater since a portion of the osmotic pressure in the cell is established by means of organic osmolytes. However, at 25°C a salinity change from 35 to about 30 (cellular interior) only has a minor impact on the diffusion coefficient for oxygen (S = 30-35: D = 2.2813-2.2593×10^{-5} cm^2 s^{-1}, Ramsing and Gundersen, 1994). Our assumption therefore is that the diffusion coefficient of seawater ($D = D_w$ = 2.2637×10^{-9} m^2 s^{-1}, Ramsing and Gundersen, 1994) is the same for the water in the cell and the surrounding water. Integrating and solving equation (2) for $\rho(r) = \rho$ yields

$$C(r)=C_b-\frac{\rho}{6D_w}\left(b^2-r^2\right), \tag{3}$$

where $C_b = C(r = b)$ is the concentration in the sphere at the inner side of the membrane and D_w is the diffusion coefficient in water. To determine C_b we consider stationary diffusion through the membrane of thickness $h = a – b$ and with a permeability P for O_2. It is assumed that there is no O_2 consumption in the membrane region. Hence in the region $b \leq r \leq a$, equation (2) is integrated for $\rho(r)$ = 0. With the total flux of O_2 through the membrane F (unit: mol O_2 s^{-1}), integration of equation (2) gives the following expression for C_b as a function of the concentration at the cell surface, $C_a = C(r = a)$,

$$C_b = C_a - \frac{F}{4\pi D_m}\frac{a-b}{ab}, \qquad (4)$$

where D_m is the diffusion coefficient in the membrane and F equals the total O_2 consumption in the region $0 \leq r \leq b$, which is given by the respiration rate per cell, i.e. $F = \frac{4}{3}\pi b^3 \rho$. We consider the situation where the thickness of the membrane $h = a - b$ is small in proportion to the radius b. In this case $ab \approx b^2$, and equation (4) takes the form

$$C_b = C_a - \frac{F}{4\pi b^2}\frac{h}{D_m} = C_a - \frac{F}{4\pi b^2}\frac{1}{P} = C_a - \frac{\rho}{3}\frac{b}{P}. \qquad (5)$$

To determine C_a we have to find the stationary concentration profile in the cell environment by solving equation (2) for $\rho(r) = 0$ in the region $r \geq a$. With the external concentration $C(r \to \infty) = C_0$, integration of equation (2) yields

$$C(r) = C_0 - \frac{F}{4\pi D_w}\frac{1}{r} = C_0 - \frac{\rho}{3D_w}\frac{b^3}{r}, \qquad (6)$$

where C_a follows for $r = a \approx b$. Replacing this value for C_a in equation (5) and the result for C_b in equation (3) gives

$$C(r) = C_0 - \frac{\rho b^2}{3D_w} - \frac{\rho b}{3P} - \frac{\rho}{6D_w}\left(b^2 - r^2\right), \qquad (7)$$

which is the equation for the concentration profile in the interior of the cell. The strongest decline of $C(r)$ occurs across the membrane of permeability P. Inside the cell the concentration profile is a very flat parabola with the minimum of O_2 concentration at $r = 0$. When $C(r)$ is zero at $r = 0$, it follows from equation (7) the permeability for O_2 (values in Table 3):

$$P = \frac{2\rho b D_w}{6C_0 D_w - 3\rho b^2} \qquad (8)$$

The membrane permeability for CO_2 follows from the permeability for O_2 by multiplication with the factor 0.7961 (Ramsing and Gundersen, 1994). Using the calculated permeabilities for O_2 and the measured net O_2 production, it follows from equation (7) (for production: ρ is replaced by $-\rho$) the intracellular O_2 concentration (values in Table 4):

$$C(r=0) = C_0 + \left(\frac{b^2}{2D_w} + \frac{b}{3P}\right)\rho. \quad (9)$$

List of abbreviations

R	cell base surface radius
l	cell length
b	inner radius of membrane
a	outer radius of membrane
h	membrane thickness
ρ	intensity of O_2 consumption (production: $-\rho$)
D	diffusion coefficient of O_2
r	radial distance from the centre of the cell
C(r)	O_2 concentration as a function of r
C_a	O_2 concentration at the cell surface
C_b	O_2 concentration at the inner side of the membrane
D_w	diffusion coefficient of O_2 in water
P	membrane permeability for O_2
F	total flux of O_2 through the membrane
D_m	diffusion coefficient of O_2 in the membrane

References

Damm E, Thoms S, Beszczynska-Möller A, Nöthig EM, Kattner G. 2015. Methane excess production in oxygen-rich polar water and a model of cellular conditions for this paradox. *Polar Science* **9(3):** 327–334.

Ramsing N, Gundersen J. 1994. *Seawater and Gases: Tabulated physical parameters of interest to people working with microsensors in marine systems*, Techn Rep MPI Mar Microbiology Bremen. [WWW document] URL http://www.unisense.com/files/PDF/Diverse/Seawater%20&%20Gases%20table.pdf [accessed 2 October 2018].

Wolf-Gladrow D, Riebesell U. 1997. Diffusion and reactions in the vicinity of plankton: A refined model for inorganic carbon transport. *Marine Chemistry* **59**: 17-34.
